# Effects of social gaze on visual-spatial imagination

**DOI:** 10.3389/fpsyg.2014.00671

**Published:** 2014-07-04

**Authors:** Heather Buchanan, Lucy Markson, Emma Bertrand, Sian Greaves, Reena Parmar, Kevin B. Paterson

**Affiliations:** ^1^School of Medicine, University of Nottingham Nottingham, UK; ^2^Department of Psychology, University of Cambridge Cambridge, UK; ^3^Institute of Criminology, University of Cambridge Cambridge, UK; ^4^School of Psychology, University of Leicester Leicester, UK

**Keywords:** visual-spatial imagery, eye-closure, gaze aversion, social interaction

## Abstract

Previous research suggests that closing one’s eyes or averting one’s gaze from another person can benefit visual-spatial imagination by interrupting cognitive demands associated with face-to-face interaction (Markson and Paterson, 2009). The present study further investigated this influence of social gaze on adults’ visual-spatial imagination, using the matrix task (Kerr, 1987, 1993). Participants mentally kept track of a pathway through an imaginary 2-dimensional (2D) or 3-dimensional (3D) matrix. Concurrent with this task, participants either kept their eyes closed or maintained eye contact with another person, mutual gaze with a person whose eyes were obscured (by wearing dark glasses), or unreciprocated gaze toward the face of a person whose own gaze was averted or whose face was occluded (by placing a paper bag over her head). Performance on the 2D task was poorest in the eye contact condition, and did not differ between the other gaze conditions, which produced ceiling performance. However, the more difficult 3D task revealed clear effects of social gaze. Performance on the 3D task was poorest for eye contact, better for mutual gaze, and equally better still for the unreciprocated gaze and eye-closure conditions. The findings reveal the especially disruptive influence of eye contact on concurrent visual-spatial imagination and a benefit for cognitively demanding tasks of disengaging eye contact during face-to-face interaction.

## INTRODUCTION

In situations involving interlocutory interactions, people often spontaneously close their eyes or look away from the interlocutor, particularly when asked difficult or probing questions (e.g., Glenberg et al., 1998; Doherty-Sneddon et al., 2002; Doherty-Sneddon and Phelps, 2005). Indeed, evidence that children are better at answering questions when their gaze is averted has led researchers to propose that children should use gaze aversion techniques in classroom settings to enhance learning (see, e.g., Doherty-Sneddon et al., 2001, 2002; Doherty-Sneddon and Phelps, 2005; Phelps et al., 2005).

Evidence for spontaneous eye-closure or gaze-avoidance when cognitive demands are particularly high is widely documented in observational studies of interlocutory interactions (typically between adults). For example, Kendon (1967) found that in filmed conversations participants averted their gaze for longer periods when speaking than when listening. Similarly, Ehrlichman (1981) observed that individuals who interacted with an interviewer on a video screen looked away from the screen more often when engaging in thinking or speaking than when listening to the interviewer. Both observations are consistent with individuals spontaneously averting their gaze to reduce environmental distraction when cognition is more demanding (e.g., thinking or producing speech compared to comprehension). Also consistent with this view, Beattie (1981) found in another observational study that looking continuously at an interviewer’s face interfered with the production of spontaneous speech and suggested that emotional arousal brought about by eye contact with an interlocutor can disrupt the formulation of responses to questions.

Other research has used experimental methods to more fully reveal the benefits of eye-closure and gaze aversion for cognition. For instance, Glenberg et al. (1998) found that performance answering general knowledge and mathematics questions was particularly impaired when participants gazed at an experimenter’s face and impaired to a lesser extent when they gazed at various visual stimuli, compared to when they closed their eyes. Glenberg et al. took these findings to indicate that gaze aversion is at least partly an effort to control cognitive load, whereby an individual averts their gaze in order to avoid input from environmental stimuli that may be irrelevant but disruptive to the task they are attempting to perform. Studies by Doherty-Sneddon et al. (2000, 2001, 2002), Doherty-Sneddon and Phelps (2005), Phelps et al. (2005) showed similar effects of gaze aversion on children’s performance on question-answering tasks. Moreover, other research that has focused on the benefits of eye-closure for episodic memory shows that recall is better when an eye-witness closes their eyes (Perfect et al., 2008, 2011; Vredeveldt et al., 2011, 2012, 2013; Vredeveldt and Penrod, 2013). Similar benefits of eye-closure for episodic memory have also been observed in an experiment in which participants had to recall images presented as part of an earlier task, either with their eyes closed or while viewing a distracting visual stimulus (Wais et al., 2010).

These benefits of eye-closure and gaze aversion are often considered from the perspective of models of dual-task performance. According to this approach, memory retrieval and environmental monitoring are competing tasks that might either be conducted simultaneously (i.e., as dual tasks) or, when one task is particularly demanding, conducted sequentially as tasks that can be switched between. Accordingly, when cognitive task demands are high, it may be beneficial to reduce environment demands by closing one’s eyes or averting one’s gaze from external stimuli (see, e.g., Glenberg, 1997). However, eye-closure and gaze aversion may offer either a modality-specific benefit (and so benefit only aspects of visual processing) or more general, cross-modal benefits for cognition (for further discussion, see, e.g., Perfect et al., 2008). For example, the view that eye-closure provides a general enhancement to memory functioning is consistent with findings showing that closing one’s eyes can improve memory for auditory as well as visual information (Perfect et al., 2008, 2011).

However, much research on the benefits of eye-closure and gaze aversion has been inspired by the multi-component model of working memory (e.g., Baddeley and Hitch, 1974; Baddeley, 1986), and often presumed that benefits will be modality-specific. A basic assumption of this model is that working memory is fractionated into two modality-specific subsystems (the visuospatial sketchpad and the phonological loop) and a multimodal subsystem (the episodic buffer), each of which are supervised by a central executive system. Dual task research has shown that the visual and phonological subsystems are subject to modality-specific interference. This, for example, entails that a concurrent secondary visual-spatial task, such as tapping out a specified pattern, will interfere with visual but not auditory memory processes (e.g., Brooks, 1968). Consequently, based on this approach, it is often argued that the disruptive influence of visual input, including that provided by eye contact with another person, is due to interference with modality-specific processing of visual information by the visual-spatial sketchpad (e.g., Wagstaff et al., 2004; Vredeveldt et al., 2011). Accordingly, eye closure or gaze aversion will specifically benefit the processing of visual-spatial information by eliminating or reducing visual interference from the environment. Additional support comes from research showing that eye-closure can enhance visual imagery (Caruso and Gino, 2011); which in turn has been shown to improve memory recall (e.g., Paivio, 1969, 1971; Jonides et al., 1975).

Doherty-Sneddon et al. (2001) conducted several experiments that more directly assessed the influence of distracting visual stimuli on the processing of visual-spatial information. These studies primarily were conducted with child participants and used various visual-spatial tasks, including the Corsi Block task (Corsi, 1972). In this task, an experimenter taps out a sequence on a set of identical spatially separated blocks and, after a short retention interval, the participant is required to reproduce this sequence. This enables the experimenter to assess the accuracy of recall for increasingly long spatial sequences. The findings were clearest for tasks, like the Corsi Block task, that included a memory component. These showed that performance was particularly poor if, during the retention interval, participants looked at someone’s face or watched a complex visual stimulus, but better if they averted their gaze (by looking at the floor) or closed their eyes. However, because these tasks included a memory component, it is unclear whether the disruptive influence of distracting visual stimuli is restricted to memory for visual-spatial information or can also affect concurrent processing of this information.

Consequently, Markson and Paterson (2009) used the matrix task (Kerr, 1987, 1993) to specifically assess the effects of distracting visual stimuli on the concurrent processing of visual-spatial information. The matrix task is a path visualization task, typically used to assess the capacity for visualizing spatial information (Kerr, 1987, 1993; Fiore et al., 2011; see also Attneave and Curlee, 1983; Diwadkar et al., 2000; Lyon et al., 2008). Participants in this task are required to mentally keep track of pathways through imaginary matrices, which can vary in complexity. These matrices typically are formed from either a 2D array of squares or a 3D cube. In a typical trial, the participant is instructed to imagine a particular matrix and is informed of the starting point of a pathway through this matrix. The direction of each successive step in the pathway is then verbally described and, at the end of the trial, the participant is required to identify its end-point, and the accuracy of their response is recorded. A major advantage of this task is that it provides an assessment of the accuracy of the visualization of this pathway without testing recall, as the participant is only required to remember the end-point and not the full pathway.

Following Kerr (1987, 1993), Markson and Paterson (2009) manipulated task difficulty by employing 2D (i.e., 3 × 3) and 3D (i.e., 3 × 3 × 3) matrices. In two experiments, performance on these matrices was compared across trials in which participants engaged concurrently in different gaze behaviors. Participants either maintained eye contact with an experimenter, kept their eyes closed, or gazed continuously at a blank computer screen or one displaying a static visual image (i.e., a picture of a sunset or an upright or inverted photograph of the experimenter) or a dynamic visual stimulus (i.e., a silent video clip). The results showed that performance on both 2D and 3D tasks was poorer when participants maintained eye contact with the experimenter than in either the eye-closure condition or the other stimulus viewing conditions, which did not differ. Markson and Paterson took this to show that maintaining eye contact with another person can impair concurrent visualization of spatial information, whereas closing one’s eyes or averting one’s gaze from that person, by viewing a blank computer screen or a static or dynamic visual stimulus, does not. In line with Kerr’s earlier findings (see also Fiore et al., 2011), performance was poorer for 3D than 2D matrices, but matrix complexity did not modulate the effects of eye-closure or averted gaze on task performance.

These experiments provide clear evidence that eye-contact with another person can disrupt visual-spatial imagination. However, it is unclear whether this disruption occurs only with eye contact or is also observed for other forms of social gaze. Various forms of social gaze can occur in social situations (e.g., Kleinke, 1986). This includes eye contact with another person, but also includes mutual gaze, in which two individuals gaze at each other’s faces without making eye contact. It is also possible to gaze upon another person while not making eye contact or engaging in mutual gaze. The question addressed in the present research is whether these different forms of social gaze produce similar disruption to visual-spatial imagination. There is considerable evidence that faces provide important social information, but it is also widely argued that the eyes more than other facial features primarily convey this information (for a review, see Itier and Batty, 2009). Consequently, eye contact may be more cognitively demanding than other forms of social gaze, including mutual gaze and unreciprocated gaze on an individual.

Accordingly, to investigate this issue further, the present research used the matrix task to assess the influence of various forms of social gaze on the visualization of spatial information. As in the original Markson and Paterson (2009) study, we examined the influence of eye-closure and eye contact with another person on task performance. But, in addition, we introduced several novel social gaze conditions. For instance, we introduced a condition in which participants engaged in mutual gaze with an experimenter without making eye contact, by having the experimenter occlude their eyes by wearing dark glasses. In another condition, participants looked continuously toward an experimenter’s face but were unable to make eye contact or engage in mutual gaze because the experimenter had averted her own gaze. Finally, we included a condition in which participants looked continuously toward the experimenter’s face but were unable to make eye contact or engage in mutual gaze, or even view that person’s face, because the experimenter had placed a paper bag over her head.

The logic of these additional social gaze conditions was straightforward. If cognitive demands associated with eye contact with another person are especially disruptive to visual-spatial imagination, performance on the matrix task should be impaired most in the eye contact condition. If mutual gaze also disrupts visualization, but to a lesser degree, performance should be better, in comparison with the eye contact condition, when only mutual gaze is possible. Moreover, performance should be better still when gaze is not reciprocated and therefore neither eye contact nor mutual gaze is possible (and this may be further enhanced by the occlusion of the experimenter’s face when she has a bag over her head). Eye-closure should also show better performance than either eye contact or mutual gaze, but it remains to be seen whether performance differs between eye-closure and unreciprocated gaze. Both 2D and 3D matrices were used in the present research, in order to determine if standard effects of matrix complexity are observed (i.e., performance should be better for 2D than 3D tasks), and to ascertain if the influence of social gaze on the visualization of spatial information varies with matrix complexity.

## MATERIALS AND METHODS

### PARTICIPANTS

Thirty undergraduate psychology students from the University of Leicester took part in the experiment in exchange for course credits.

### DESIGN

The experiment manipulated two within-participants independent variables. The first was the number of dimensions for the matrix and had two levels: 2D (3 × 3) and 3D (3 × 3 × 3) matrices. The second independent variable was the gaze condition, which had five levels: participants either kept their eyes closed, maintained eye contact with an experimenter whose eyes were fully visible, maintained mutual gaze with an experimenter whose eyes were obscured by wearing dark glasses, or gazed continuously toward the face of an experimenter who either averted their own gaze or whose face was occluded by placing a paper bag over her head. The dependent variable was the number of correct responses in the matrix task (i.e., responses that accurately identified the correct end-point of a pathway).

### MATERIALS AND APPARATUS

The 2D matrix was drawn in black ink on white cardboard. Each square of the matrix was 4 cm^2^. The 3D matrix was built from wooden blocks, each measuring 4 cm^3^. The pathways were based on those used by Markson and Paterson (2009). Each pathway had a designated starting square or block and comprised seven statements expressing a sequence of one unit moves in either up, down, left and right directions for the 2D matrix and also forward and backward directions for the 3D matrix. No directional term appeared more than twice consecutively in each sequence. Audio-recordings of these directional statements were played to participants and served to provide directional instructions in each gaze condition. The directional statements were recorded with an interval rate of 0.5 s, read to the time of a metronome, as this was the presentation rate at which Kerr (1993) observed clear differences in performance between 2D and 3D matrices.

### PROCEDURE

Participants took part individually and were told they were taking part in a study of perceptual processing. Written instructions on how to complete the task were given to participants, and participants took part in two practice trials, one for a 2D matrix and one for a 3D matrix, before beginning the experiment. Participants were instructed to maintain eye contact with an experimenter in the eye contact condition, to maintain gaze toward the experimenter’s eyes in the mutual gaze condition, and to maintain gaze in the direction of the experimenter’s face in the unreciprocated gaze conditions. In the eye-closure condition, participants were instructed to keep their eyes firmly closed throughout the trial. A second experimenter checked compliance with these instructions and repeated the instructions between trials as a reminder if this proved necessary. Participants stood 1.5 m from the experimenter in each social gaze condition.

At the beginning of each trial, the experimenter showed either the 2D or 3D matrix to the participant and indicated the matrix’s starting point. The matrix was then removed from the participant’s view, and the directional instructions for that trial were played to the participant. Throughout each trial, the experimenter remained silent, stationary, and expressionless. At the end of the trial, the experimenter showed the matrix to the participant and asked them to indicate the final square or block in the pathway that had been described. The participant’s response was then recorded. Each participant performed four 2D and four 3D trials in each social gaze condition, in five separate blocks that were counterbalanced for order across participants. The experiment lasted approximately 40 minutes per participant.

## RESULTS

**FIGURE 1:**
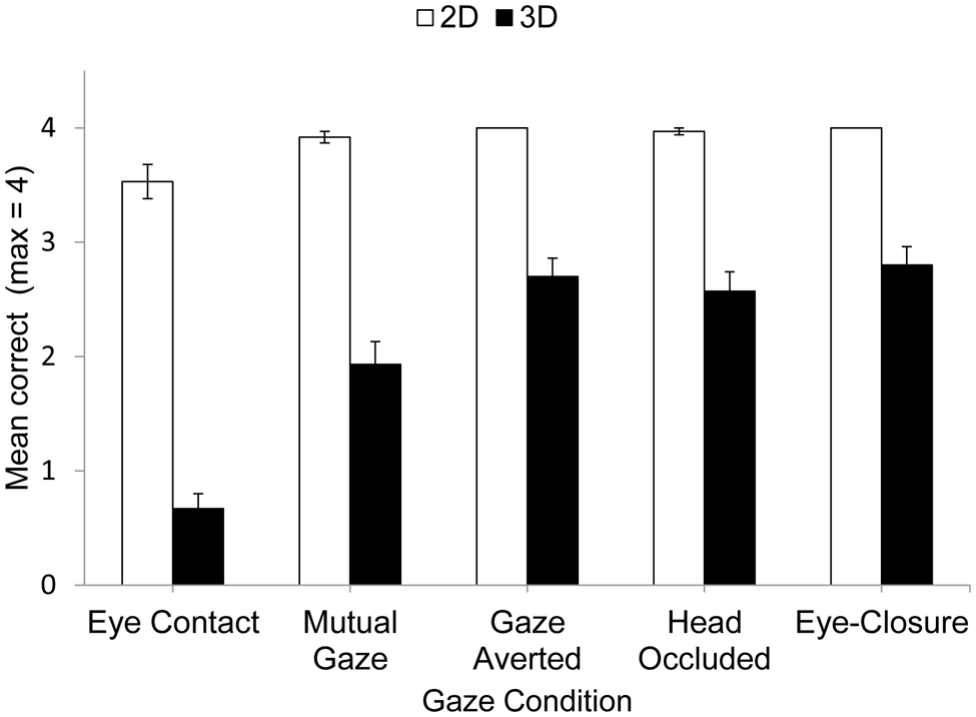
**Mean correct responses in 2D and 3D matrix tasks.** Bars correspond to standard errors.

The mean performance accuracy for 2D and 3D matrices in each social gaze condition is shown in **Figure 1**.

Performance on the matrix task was analyzed using 2 (matrix complexity) × 5 (gaze condition) analysis of variance (ANOVA), and the Greenhouse–Geisser correction was used where appropriate. The analysis revealed a significant main effect of Matrix Complexity, *F*(1,29) = 272.10, *p*< 0.001, $\eta_{p}^{2}$ = 0.90. This was due to participants making more correct responses for 2D matrices (*M*= 3.9) than 3D matrices (*M*= 2.1). This replicated earlier findings (Kerr, 1987, 1993; Markson and Paterson, 2009; Fiore et al., 2011). There was also a significant main effect of Gaze Condition, *F*(4,76) = 58.87, *p*< 0.001, $\eta_{p}^{2}$ = 0.67, and a significant interaction that revealed that the influence of 5Gaze Condition was modulated by Matrix Complexity, *F*(4,76) = 19.78, *p*< 0.001, $\eta_{p}^{2}$ = 0.41. This interaction was explored further using a Bonferroni-corrected *t*-test. For the 2D task, performance was poorer for eye contact compared to the other gaze conditions (eye contact vs. mutual gaze, *p* < 0.01, *d* = 0.56; eye contact vs. averted gaze, *p* < 0.01, *d* = 0.57; eye contact vs. bag over head, *p* < 0.01, *d* = 0.50; eye contact vs. eye-closure, *p* < 0.01, *d* = 0.56). No other differences were significant (*p* > 0.15, *d* < 0.30).

For the more difficult 3D task, performance also was poorer for eye contact compared to the other gaze conditions (eye contact vs. mutual gaze, *p* < 0.001, *d* = 1.08; eye contact vs. averted gaze, *p* < 0.001, *d* = 2.04; eye contact vs. bag over the head, *p* < 0.001, *d* = 1.57; eye contact vs. eye-closure, *p* < 0.001, *d* = 1.98). In addition, performance was poorer for mutual gaze compared to either averted gaze (*p* < 0.01, *d* = 0.73), occluding the experimenter’s face by placing a bag over her head (*p* < 0.001, *d* = 0.63), or eye-closure (*p* < 0.001, *d* = 0.89). No other differences were significant (*p* > 0.05, *d* < 0.04). The indication, therefore, is that maintaining eye contact with another person is especially disruptive to accurate mental visualization of a pathway through a matrix. Moreover, mutual gaze is also disruptive to accurate visualization of this pathway, but less so than eye contact. Finally, social gaze conditions in which gaze is not reciprocated and so eye contact and mutual gaze are not possible are no more disruptive to this visualization than eye-closure^1^.

## DISCUSSION

The results of this experiment were very clear. In line with previous studies, more correct responses were produced for 2D than 3D matrices (Kerr, 1987, 1993; Markson and Paterson, 2009; Fiore et al., 2011). Consequently, participants in the present experiment showed similar sensitivity to matrix complexity as participants in previous research. In addition, as in previous research which used the matrix task to investigate effects of eye-closure and gaze aversion on visual-spatial imagination (Markson and Paterson, 2009), maintaining eye contact with another person disrupted an individual’s ability to keep track of a pathway through an imaginary matrix in both 2D and 3D versions of the task. Performance on 2D matrices in the other social gaze conditions did not differ and was at ceiling. However, performance in the 3D matrix task revealed important differences between social gaze conditions. For this more difficult matrix task, performance was poorest for the eye contact condition, better for mutual gaze, and better still in conditions in which gaze was not reciprocated and so neither eye contact nor mutual gaze was possible. Interestingly, the unreciprocated gaze conditions did not differ in performance, indicating that occluding the experimenter’s face did not bring additional benefits to performance. Moreover, both unreciprocated gaze conditions produced as good performance as eye-closure. Consequently, the indication from the present findings is that maintaining eye contact with another individual is singularly disruptive to visual-spatial imagination. Mutual gaze is also disruptive to visual-spatial imagination but less so than eye contact. Finally, other forms of social gaze that do not require reciprocation (i.e., maintaining gaze on someone who has averted their own gaze or whose face is occluded) produced the same level of performance as eye-closure, and so appear not to disrupt the visualization of spatial information.

The present findings are in line with previous findings by Markson and Paterson (2009) who observed that performance on 2D and 3D matrices was especially disrupted by maintaining eye contact with another person, but that performance was largely unaffected by other forms of visual stimuli. In particular, it had previously been suggested that processing faces requires visual-spatial working memory resources and that averting gaze from a person’s face, or closing one’s eyes, can preserve these working memory resources for use in other cognitive tasks (Doherty-Sneddon et al., 2001). Consequently, viewing an image of a person’s face might be expected to be disruptive to visual-spatial processing. However, Markson and Paterson found that gazing upon either an upright image of the experimenter’s face or an inverted image of the experimenter’s face (which might be expected to be less disruptive) produced similar performance to eye-closure. It therefore appeared that demands associated with processing the image of a face did not interfere with the concurrent visualization of spatial information. The present findings expand on these previous findings by showing that face processing may only be disruptive to a visualization task when a live person is involved and this is accompanied by eye contact or mutual gaze.

Such findings are not particularly surprising given the substantial evidence for the special status of eye contact and mutual gaze in social situations. Indeed, there is abundant evidence that looking at another person’s face, and particularly their eyes, provides a wealth of complex cognitive information. This can include information about the other person’s direction of gaze and their emotional and mental states, but eye contact also plays an important role in regulating social interaction by, for example, providing cues to turn-taking during conversation (for a review see, Frischen et al., 2007). Looking at the eyes of another person has also been shown to elicit a host of social cognitive and affective responses, including heightened self-awareness and a sense of intimacy (e.g., Argyle, 1981; Kleinke, 1986). Indeed, physiological evidence shows that eye contact in particular increases skin conductance and produces higher scores on subjective self-assessments of emotional arousal and valence compared to averted gaze or looking at a picture of a person (Hietanen et al., 2008; Akechi et al., 2013). Moreover, recent electrophysiological research has revealed differences in the neural response to viewing another person in the same room compared to viewing that person on a computer screen or in a photograph, and that viewing a live face with direct gaze is processed more intensely than a face with averted gaze or closed eyes (Pönkänen et al., 2011). Consequently, it seems likely that the especially disruptive effect of eye contact on visual-spatial imagination in the present experiment and in the earlier research by Markson and Paterson (2009) is related to the heightened cognitive and social demands associated with maintaining eye contact with a live person. These demands are lessened in mutual gaze conditions and appear to be effectively eliminated when both eye contact and mutual gaze are prevented.

By comparison with previous studies of memory for visual-spatial information (Doherty-Sneddon et al., 2001), there was no evidence for a more extensive influence of visual interference on task performance. Doherty-Sneddon et al. (2001) found that the performance of child participants who performed visual-spatial memory tasks was poorer in conditions in which they viewed a dynamic visual stimulus compared to when they closed their eyes or averted their gaze (by looking at the floor) in the interval between viewing the test stimuli and providing a response. However, there was no indication from the experiments by Markson and Paterson (2009) that viewing either static or dynamic images is any more disruptive to visual-spatial imagination than averting one’s gaze or closing one’s eyes. Similarly, the present experiments show no benefit for eye-closure over situations in which participants gaze at another person without making eye contact or engaging in mutual gaze. Thus, it appears that visual input is not a significant source of interference in the matrix task, but social interaction involving either eye contact or mutual gaze is.

The particular advantage of the matrix task is that it provides an assessment of visual-spatial processing separate from memory for this information. Consequently, findings obtained with the matrix task may differ from those obtained with other tasks because it provides an assessment of effects associated with visual-spatial imagery rather than the retention of this information or its retrieval from memory. A further important difference is that whereas the present research (and the original experiments by Markson and Paterson, 2009) investigated effects of eye-closure and gaze aversion on visualization of spatial information by adult participants, the earlier work by Doherty-Sneddon et al. (2001) focused on these processes in children. Consequently, further research is required to determine if the contrast in the findings obtained in these studies reflect this difference in the age of the participants.

An important additional advantage of the matrix task over other tests of visual-spatial processing is that it provides an effective means of assessing the influence of the social environment on visual-spatial imagery. Indeed, while the present experiment provides insight into the influence of social demands on task performance, various factors remain to be investigated. For instance, a factor which may be particularly important is the social distance between the participant and the experimenter when performing the matrix task. Indeed, pilot data from our laboratory suggest that effects of eye contact are mediated by the physical proximity of the participant and the experimenter, and that effects of eye contact may be obtained only at standard social distances (i.e., when the participant and the experimenter are approximately 1 m apart, e.g., Argyle and Dean, 1965). These pilot data suggest that the influence of eye contact may be disrupted at closer distances and dissipate when the participant and experimenter are further apart (i.e., 3 m or more apart), although further research is required to fully establish these effects.

Markson and Paterson (2009) also argued that a particularly important avenue of research might involve assessing effects of individual differences in social anxiety or shyness on task performance, as individuals scoring high on these characteristics may show heightened sensitivity to social demands when eye contact is made compared to when gaze is averted or the individual closes their eyes (see also Moukheiber et al., 2012). Indeed, if eye-closure or gaze aversion benefit cognition by individuals who suffer acutely from social anxiety or shyness, it may be advantageous to encourage these individuals to adopt these techniques in relevant settings (e.g., in the classroom). However, it is also important to note that negative social judgments frequently are made of individuals who avert their gaze or turn away from an interlocutor (e.g., Larsen and Shackelford, 1996), especially as people who avert their gaze are often perceived as deceptive by others (for discussion see, e.g., Vrij and Semin, 1996; Mann et al., 2002; Einav and Hood, 2008). Finally, as noted already, the present studies used only adult participants. Consequently, an obvious future direction for this research would be to examine how children perform in the matrix task. Such experiments could include manipulations of social gaze or social proximity and would have the potential to reveal development changes in the influence of the social situation on the performance of cognitively demanding tasks.

## Conflict of Interest Statement

The authors declare that the research was conducted in the absence of any commercial or financial relationships that could be construed as a potential conflict of interest.

## Footnotes

1 Non-parametric analyses of the effects of the different gaze conditions on task performance were conducted separately for 2D and 3D tasks using the Friedman Test (Siegel and Castellan, 1988). This confirmed there was a significant effect of gaze condition for the 2D task, χ^2^(4) = 27.06, *p* < 0.001, and for the 3D task, χ^2^(4) = 66.12, *p* < 0.001. *Post hoc* analyses were conducted using a Bonferroni-corrected Wilcoxon Test. For the 2D task, performance was poorer in the eye contact condition compared to the other gaze conditions (*p*s < 0.01). For the 3D task, performance was poorer in the eye contact condition compared to the mutual gaze condition (*p* < 0.01), and for the mutual gaze condition compared to the unreciprocated gaze and eye-closure conditions (*p*s < 0.01). Thus, the principle findings of this experiment were upheld using these non-parametric statistical analyses.

## References

[B1] Akechi H. Senju A. Uibo H. Kikuchi Y. Hasegawa T. Hietanen J. K. (2013). Attention to eye contact in the West and East: autonomic responses and evaluative ratings. *PLoS ONE* 8:e59312 10.1371/journal.pone.0059312 PMC3596353 23516627

[B2] Argyle M. (1981). *Bodily Communication.* London, England: Methuen.

[B3] Argyle M. Dean J. (1965). Eye-contact, distance, and affiliation. *Sociometry* 28 289–304. 10.2307/2786027 14341239

[B4] Attneave F. Curlee T. E. (1983). Locational representation in imagery: a moving spot task. *J. Exp. Psychol. Hum. Percept. Perform.* 9 20–30. 10.1037/0096-1523.9.1.20 6220121

[B5] Baddeley A. D. (1986). *Working Memory.* Oxford: Oxford University Press.

[B6] Baddeley A. D. Hitch G. J. (1974). “Working memory,” in *Recent Advances in Learning and Motivation,* Vol. 8 ed.Bower G. A. (New York: Academic Press) 47–89.

[B7] Beattie G. W. (1981). A further investigation of the cognitive interference hypothesis of gaze patterns during conversation. *Br. J. Soc. Psychol.* 20 243–148. 10.1111/j.2044-8309.1981.tb00493.x

[B8] Brooks L. (1968). Spatial and verbal components in the act of recall. *Can. J. Psychol.* 22 349–368. 10.1037/h0082775

[B9] Caruso E. Gino F. (2011). Blind ethics: closing one’s eyes polarizes moral judgments and discourages dishonest behavior. *Cognition* 118 280–285. 10.1016/j.cognition.2010.11.008 21145538

[B10] Corsi P. M. (1972). Human memory and the medial temporal region of the brain. *Dissert. Abstracts Int.* 34 891B.

[B11] Diwadkar V. A. Carpenter P. A. Just M. A. (2000). Collaborative activity between parietal and dorsolateral prefrontal cortex in dynamic spatial working memory revealed by fMRI. *Neuroimage* 12 85–99. 10.1006/nimg.2000.0586 10875905

[B12] Doherty-Sneddon G. Bonner L. Bruce. (2001). Cognitive demands of face monitoring: evidence for visuospatial overload. *Mem. Cognit.* 29 909–919. 10.3758/BF03195753 11820750

[B13] Doherty-Sneddon G. Bruce V. Bonner L. Longbotham S. Doyle C. (2002). Development of gaze aversion as disengagement from visual information. *Dev. Psychol.* 38 438–445. 10.1037/0012-1649.38.3.438 12005386

[B14] Doherty-Sneddon G. McAuley S. Bruce V. Langton S. Blokland A. Anderson A. H. (2000). Visual signals and children’s communication: negative effects on task outcome. *Br. J. Dev. Psychol.* 18 595–608. 10.1348/026151000165878

[B15] Doherty-Sneddon G. Phelps F. G. (2005). Gaze aversion: a response to cognitive or social difficulty? *Mem. Cognit.* 33 727–733. 10.3758/BF03195338 16248336

[B16] Ehrlichman H. (1981). From gaze aversion to eye-movement suppression: an investigation of the cognitive interference explanation of gaze patterns during conversation. *Br. J. Soc. Psychol.* 20 233–241. 10.1111/j.2044-8309.1981.tb00492.x

[B17] Einav S. Hood B. M. (2008). Tell-tale eyes: children’s attribute of gaze aversion as a lying cue. *Dev. Psychol.* 44 1655–1667. 10.1037/a0013299 18999328

[B18] Fiore F. Borella E. Mammarella I. C. Cornoldi C. (2011). Mental imagery in a visuospatial working memory task and modulation of activation. *J. Cogn. Psychol.* 23 52–59. 10.1080/20445911.2011.454497

[B19] Frischen A. Bayliss A. P. Tipper S. P. (2007). Gaze cueing of attention: visual attention, social cognition, and individual differences. *Psychol. Bull.* 133 694–724. 10.1037/0033-2909.133.4.694 17592962 PMC1950440

[B20] Glenberg A. M. (1997). What memory is for. *Behav. Brain Sci.* 20 1–55. 10096994 10.1017/s0140525x97000010

[B21] Glenberg A. M. Schroeder J. L. Robertson D. A. (1998). Averting the gaze disengages the environment and facilitates remembering. *Mem. Cognit.* 26 651–658. 10.3758/BF03211385 9701957

[B22] Hietanen J. K. Leppanen J. M. Peltola M. J. Linna-Aho K. Ruuhiala H. J. (2008). Seeing direct and averted gaze activates the approach-avoidance motivational brain systems. *Neuropsychologia* 46 2423–2430. 10.1016/j.neuropsychologia.2008.02.029 18402988

[B23] Itier R. J. Batty M. (2009). Neural bases of eye and gaze processing: the core of social cognition. *Neurosci. Biobehav. Rev.* 33 843–863. 10.1016/j.neubiorev.2009.02.004 19428496 PMC3925117

[B24] Jonides J. Kahn R. Rozin P. (1975). Imagery instructions improve memory in blind subjects. *Bull. Psychon. Soc.* 5 424–426. 10.3758/BF03333288

[B25] Kendon A. (1967). Some functions of gaze direction in social interaction. *Acta Psychol.* 26 22–63. 10.1016/0001-6918(67)90005-4 6043092

[B26] Kerr N. H. (1987). Locational representation in imagery: the third dimension. *Mem. Cognit.* 15 521–530. 10.3758/BF03198387 3695947

[B27] Kerr N. H. (1993). Rate of imagery processing in two versus three dimensions. *Mem. Cognit.* 21 467–476. 10.3758/BF03197178 8350738

[B28] Kleinke C. L. (1986). Gaze and eye contact: a research review. *Psychol. Bull.* 100 78–100. 10.1037/0033-2909.100.1.78 3526377

[B29] Larsen R. J. Shackelford T. K. (1996). Gaze avoidance: personality and social judgments of people who avoid direct face-to-face contact. *Pers. Individ. Dif.* 21 907–917. 10.1016/S0191-8869(96)00148-1

[B30] Lyon D. R. Gunzelmann G. Gluck K. E. (2008). A computational model of spatial visualization capacity. *Cogn. Psychol.* 57 122–152. 10.1016/j.cogpsych.2007.12.003 18314098

[B31] Mann S. Vrij A. Bull R. (2002). Suspects, lies, and videotape: an analysis of authentic high-task liars. *Law Hum. Behav.* 26 365–376. 10.1023/A:1015332606792 12061624

[B32] Markson L. Paterson K. B. (2009). Effects of gaze aversion on visual-spatial imagination. *Br. J. Psychol.* 100 553–563. 10.1348/000712608X371762 19021925

[B33] Moukheiber A. Rautureau G. Perez-Diaz F. Jouvent R. Pelissolo A. (2012). Gaze behaviour in social blushers. *Psychiatry Res.* 200 614–619. 10.1016/j.psychres.2012.07.017 22951333

[B34] Paivio A. (1969). Mental imagery in associative learning and memory. *Psychol. Rev.* 76 241–263. 10.1037/h0027272

[B35] Paivio A. (1971). Imagery and deep structure in the recall of English nominalizations. *J. Verbal Learning Verbal Behav.* 10 1–12. 10.1016/S0022-5371(71)80086-5

[B36] Perfect T. J. Andrade J. Eagan I. (2011). Eye closure reduces the cross-modal memory impairment caused by auditory distraction. *J. Exp. Psychol. Learn. Mem. Cogn.* 37 1008–1013. 10.1037/a0022930 21417514

[B37] Perfect T. J. Wagstaff G. F. Moore D. Andrews B. Cleveland V. Newcombe S. (2008). How can we help witnesses to remember more? It’s an (eyes) open and shut case. *Law Hum. Behav.* 32 314–324. 10.1007/s10979-007-9109-5 17899340

[B38] Phelps F. G. Doherty-Sneddon G. Warnock H. (2005). Helping children think: gaze aversion and teaching. *Br. J. Dev. Psychol.* 24 577–588. 10.1348/026151005X49872

[B39] Pönkänen L. M. Alhoniemi A. Leppänen J. M. Hietanen J. K. (2011). Does it make a difference if I have an eye contact with you or with your picture? An ERP study. *Soc. Cogn. Affect. Neurosci.* 6 486–494. 10.1093/scan/nsq068 20650942 PMC3150859

[B40] Siegel S. Castellan N. J. (1988). *Nonparametric Statistics for the Behavioral Sciences* 2nd Edn. New York, NY: McGraw-Hill.

[B41] Vredeveldt A. Baddeley A. D. Hitch G. J. (2012). The effects of eye-closure and “ear-closure” on recall of visual and auditory aspects of a criminal event. *Eur. J. Psychol.* 8 284–299. 10.5964/ejop.v8i2.472

[B42] Vredeveldt A. Baddeley A. D. Hitch G. J. (2013). The effectiveness of eye-closure in repeated interviews. *Leg. Criminol. Psychol.* 10.1111/lcrp.12013 [Epub ahead of print].

[B43] Vredeveldt A. Hitch G. J. Baddeley A. D. (2011). Eye-closure helps memory by reducing cognitive load and enhancing visualisation. *Mem. Cognit.* 39 1253–1263. 10.3758/s13421-011-0098-8 21491166

[B44] Vredeveldt A. Penrod S. D. (2013). Eye-closure improves memory for a witnessed event under naturalistic conditions. *Psychol. Crime Law* 19 893–905. 10.1080/1068316X.2012.700313

[B45] Vrij A. Semin G. R. (1996). Lie experts’ beliefs about nonverbal indicators of deception. *J. Nonverbal Behav.* 20 65–80. 10.1007/BF02248715

[B46] Wagstaff G. F. Brunas-Wagstaff J. Cole J. Knapton L. Winterbottom J. Crean V. (2004). Facilitating memory with hypnosis, focused meditation, and eye closure. *Int. J. Clin. Exp. Hypnosis* 52 434–455. 10.1080/00207140490889062 15590510

[B47] Wais P. E. Rubens M. T. Boccanfuso J. Gazzaley A. (2010). Neural mechanisms underlying the impact of visual distraction on retrieval of long-term memory. *J. Neurosci.* 30 8541–8550. 10.1523/JNEUROSCI.1478-10.2010 20573901 PMC2919837

