# Erratum: Effects of social gaze on visual-spatial imagination

**DOI:** 10.3389/fpsyg.2018.01053

**Published:** 2018-06-21

**Authors:** Frontiers Production Office

**Affiliations:** Frontiers Production Office Frontiers Media SA, Lausanne, Switzerland

**Keywords:** visual-spatial imagery, eye-closure, gaze aversion, social interaction

An error in the editor name was introduced during typesetting. The correct editor name is Timothy John Hollins.

The publisher apologizes for this error and the original article has been corrected. This error does not change the scientific conclusions of the article in any way.

The original article has been updated.

